# Short Telomere Length is Associated with Aging, Central Obesity, Poor Sleep and Hypertension in Lebanese Individuals

**DOI:** 10.14336/AD.2017.0310

**Published:** 2018-02-01

**Authors:** Nathalie K Zgheib, Fatima Sleiman, Lara Nasreddine, Mona Nasrallah, Nancy Nakhoul, Hussain Isma’eel, Hani Tamim

**Affiliations:** ^1^Department of Pharmacology & Toxicology, Faculty of Medicine, American University of Beirut, Lebanon; ^2^Department of Nutrition & Food Sciences, Faculty of Agriculture and Food Sciences, American University of Beirut, Lebanon; ^3^Department of Internal Medicine, Faculty of Medicine, American University of Beirut Medical Center, Lebanon; ^4^Clinical Research Institute, Faculty of Medicine, American University of Beirut Medical Center, Lebanon

**Keywords:** Aging, Hypertension, Obesity, Relative telomere length, Sleep

## Abstract

In Lebanon, data stemming from national cross-sectional surveys indicated significant increasing trends in the prevalence of cardiovascular diseases and associated behavioral and age-related risk factors. To our knowledge, no data are available on relative telomere length (RTL) as a potential biomarker for age-related diseases in a Lebanese population. The aim of this study was to evaluate whether there is an association between RTL and demographic characteristics, lifestyle habits and diseases in the Lebanese. This was a cross-sectional study of 497 Lebanese subjects. Peripheral blood RTL was measured by amplifying telomere and single copy gene using real-time PCR. Mean ± SD RTL was 1.42 ± 0.83, and it was categorized into 3 tertiles. Older age (*P*=0.002) and wider waist circumference (WC) (*P*=0.001) were statistically significantly associated with shorter RTL. Multinomial logistic regression showed that subjects who had some level of sleeping difficulty had a statistically significantly shorter RTL when compared to those with no sleeping difficulties at all [OR (95% CI): 2.01 (1.11-3.62) in the first RTL tertile]. Importantly, statistically significantly shorter RTL was found with every additional 10 cm of WC [OR (95% CI): 1.30 (1.11-1.52) for first RTL tertile]. In addition, and after performing the multivariate logistic regression and adjusting for “predictors” of RTL, the odds of having hypertension or being treated for hypertension were higher in patients who had shorter RTL: OR (95% CI): 2.45 (1.36-4.44) and 2.28 (1.22-4.26) in the first RTL tertiles respectively with a similar trend, though not statistically significant, in the second RTL tertiles. This is the first study in Lebanon to show an association between age, central obesity, poor sleep and hypertension and RTL. It is hoped that telomere length measurement be potentially used as a biomarker for biological age and age-related diseases and progression in the Lebanese.

Telomeres are double stranded, repetitive sequences of six nucleotides (TTAGGG) that cap the ends of the linear eukaryotic chromosomes [1]. Telomeres are bound to human shelterin which is composed of six telomere-binding proteins. Both telomeres and human shelterin maintain genetic integrity, hence cellular stability, as they are important to protect the end of chromosomes from fusion [2]. In addition, they are both required to avoid the loss of coding nucleotides during each eukaryotic DNA replication, which is known as the chromosome end-replication problem due to the inability of the cell to fully replicate the 5′ end of the lagging DNA strand [3]. This replication problem results in the shortening of the telomere during each cell division till it reaches a critical length [4]. Having one or more critically short telomeres causes the cell to become senescent whereby any further cell division threatens the integrity of the coding DNA [5, 6].

Telomere length was found to shorten with age. For instance, a study including 137 individuals ranging from 0 to 104 years showed that telomere shortening increased in older individuals and in cells with rapid turnover when compared to static cells such as myocardium cells that may exhibit telomere maintenance machinery [7]. As a matter of fact, telomere shortening may predispose individuals to age-related diseases and increased mortality [8-10]. For example, shorter relative telomere length (RTL) was found to be associated with increased severity of chronic heart failure (CHF), atherosclerotic disease progression, and a 3-fold increase in risk of myocardial infarction [11, 12]. Similarly, patients with metabolic diseases such as type 2 diabetes mellitus had shorter peripheral blood RTL when compared to control subjects. Interestingly, diabetic patients with long telomeres had less vascular changes compared to those with short telomeres [13]. These findings supported using telomere as a potential biomarker not only for chronological age, but also for biological age that reflects the individual’s health status [14].

Many life style factors were also shown to affect RTL [15-17]. For example, smoking and alcohol consumption were shown to shorten telomeres in a dose-dependent manner [18, 19], while eating food rich in antioxidants such as legumes, whole grains, fish, and vegetables was shown to be associated with longer RTL [20, 21]. Nevertheless, obesity and increased body mass were associated with shortened telomeres [22]. Interestingly, stress may also cause telomere shortening, as Epel *et al*. [23] reported that women under stress had a shortening of RTL equivalent to an additional 10 years increase in their age.

In Lebanon, data stemming from national cross-sectional surveys indicated significant increasing trends in the prevalence of cardiovascular diseases and associated behavioral and age-related risk factors [24-29]. To our knowledge, some results were published in Arabs [30-33], but no data are available on RTL in a Lebanese population. This study aimed to measure RTL in a sample of Lebanese individuals, and evaluate whether there is an assocition between their RTL and demographic characteristics, lifestyle habits and diseases. This is with the aim to potentially use telomere length measurement as a biomarker for biological age and age-related diseases and progression [34].

## MATERIALS AND METHODS

The current study utilized data from an available database of a study carried out in 2014. Following is a description of the original study, as well as details of the current analyses.

### Original study

It was a cross-sectional, community-based study using multistage probability sampling, of a representative sample of 501 adult Lebanese men and women (age ≥ 18 years) residing in Greater Beirut that were recruited between February and June 2014. The objective of the study was to assess the level of Bisphenol A among the residents in Greater Beirut. The study and the current analysis were approved by the Institutional Review Board (IRB) of the American University of Beirut. The methods were carried out in accordance with the relevant guidelines and regulations, and all participants signed an informed consent form.

Face-to-face interviews, anthropometric measurements and blood withdrawal for laboratory tests were carried out by very well-trained study personnel. The questionnaire included data on: (1) demographic and socioeconomic characteristics: age, gender, marital status, education, occupation, crowding index based on number of residents and rooms in the dwelling, and monthly income bracket per family; (2) lifestyle: current smoking intake to include regular cigarettes and narghileh (a type of hookah pipe commonly smoked in Lebanon), alcohol intake, caffeine intake, physical activity (using the short version of the International Physical Activity Questionnaire (IPAQ) [35]) and questions on sleeping habits; (3) medical history for chronic diseases: such as type 2 diabetes mellitus, hypertension and dyslipidemia. Drug intake for chronic diseases was also collected.

Anthropometric measurements included: weight and height using calibrated scale and stadiometer, waist and hip circumferences using standardized protocols, and percent body fat and muscle mass using a tetrapolar electrical bioimpedance analyzer (Inbody Body Composition Analyzer, Inbody 230, InBody Co., Ltd., Seoul, Korea). Diastolic and systolic blood pressures were also measured using a calibrated classic sphygmomanometer.

Blood was drawn into 2 chemistry tubes and an EDTA tube. Whole blood in the EDTA tube was stored at -80 ºC for future DNA isolation. The chemistry tubes were centrifuged for plasma within one hour and aliquoted into 1 mL microtubes, some of which were immediately transported to the College of American Pathologists (CAP) certified laboratory of the American University of Beirut Medical Center (http://labmed.aub.edu.lb/plm/), while the rest were stored at -20 ºC and sent to the laboratory as batches depending on the needed measurements as per guidelines. These laboratory measurements included fasting glucose, hemoglobin A1C (HbA1C), insulin, C-peptide, lipid profile, cortisol, and C-reactive protein (CRP).

### Current study

Included in this study was the sample included in the original study for whom DNA was available for RTL analysis.

### Relative telomere length measurement

Total DNA was extracted from leucocytes of peripheral venous blood using Qiagen kit (Qiagen, USA) as per manufacturer guidelines, normalized to a concentration of10 ng/ul, and stored at -20 ºC until analysis. RTL was measured by amplifying telomere (tel) and single copy gene (human beta-globin-hbg) separately, using quantitative real-time polymerase chain reaction (RT-qPCR) on CFX384 Touch Real-Time PCR Detection System from BIO-RAD as described by Cawthon’s method 2002 with few adjustments made by Cawthon in 2009 [36, 37]. Primers used for telomere gene were tel c (5′-TgTTAggTATCCCTATCCCTATCCCTATCCCTA TCCCTAACA) and telg (5′- ACACTAAggTTTggg TTTgggTTTgggTTTgggTTAgTgT). As for the single copy gene primers, the following were used: hbg1 (5′- ACACTAAggTTTgggTTTgggTTTgggTTTgggTTAgTgT) and hbg 2 (5′- CACCAACTTCATCCACgTTCACC). Thermal cycling conditions for tel were: 50ºC for 2 min, 95ºC for 2 min, then two cycles of 95ºC for 15 sec, 49ºC for 15 sec, then 35 cycles of 95ºC for 15 sec, 62ºC for 10 sec, and 74ºC for 15 sec. For hbg: 50ºC for 2 min, 95ºC for 2 min, then 36 cycles of 95ºC for 15 sec and 58ºC for 1 min. RTL was calculated according to the formula described by Pfaffl 2001 to account for different plate efficiencies [38]. For the telomere and single copy gene qPCRs, 384-well plates were used and the samples were run in triplicates along with standards. Two randomly chosen DNA samples were included in every run as reproducibility controls. A no-template control was also included. Four standards were prepared from a pool of DNA samples with concentrations of 20ng/uL, 10ng/uL, 2.5ng/uL and 0.3125ng/uL respectively. Standard curves were drawn, and slopes along with the correlation coefficients (R^2^) were calculated using the CFX Manager 3.1 software. A melt curve was performed for each qPCR run to ensure reaction specificity and check for any primer-dimer formation.

### Statistical analysis

Data were entered into SPSS version 23.0 (IBM, USA) and a P <0.05 was considered to indicate statistical significance. RTL was categorized into 3 groups based on statistical grounds (tertiles). Data were computed as means ± standard deviation (SD) for continuous variables, and as numbers and percent for categorical variables.

In addition to the questions on whether patients were diagnosed with type 2 diabetes mellitus, whether they were treated for diabetes and the associated laboratory values, a “definite diabetes” variable was computed for subjects who were diagnosed with diabetes and/or both fasting blood sugar (FBS) was ≥126 mg/dL and HbA1C was ≥6.5% [39]. Similarly, for hypertension, a “definite hypertension” variable was computed for those who were diagnosed with hypertension or had an abnormal blood pressure reading upon recruitment (systolic blood pressure≥140 mm/Hg or diastolic blood pressure≥90 mm/Hg) [40]. This was not done for dyslipidemia due to complexities in the categorization of normal lipid values [41]; nevertheless, a metabolic syndrome (MetS) variable was computed based on the joint harmonized definition of the International Diabetes Federation Task Force on Epidemiology and Prevention; the National Heart, Lung, and Blood Institute; the American Heart Association; the World Heart Federation; the International Atherosclerosis Society; and the International Association for the Study of Obesity [42]. The 10-year atherosclerotic cardiovascular disease (ASCVD) was also calculated based on the American College of Cardiology and American Heart Association guideline issued in 2013. Participants with cardiovascular or cerebrovascular disease were excluded from the ASCVD analysis [43].

The associations of potential predictors (demographic variables, socioeconomic characteristics, lifestyle habits) and outcomes (chronic diseases and laboratory measurements) with RTL were first evaluated using univariate ANOVA and Chi-square test, as applicable. Afterwards, multivariate analyses were carried out to control for confounding effect when assessing these associations. More specifically, potential predictors for RTL were assessed by carrying out a stepwise multinomial logistic regression model which included demographic, socioeconomic and lifestyle variables that were found to be significantly associated with RTL in the univariate analysis. Significant predictors were then included in the final outcome analysis. Note that in this analysis, and due to the exploratory nature of the study, the associations between RTL and all potential outcomes, even if they did not show significant results in the initial univariate analysis, were assessed using stepwise logistic regression analyses. Results of the multivariate analyses are reported as odds ratio (OR) for categorical variables and Beta coefficient for continuous variables with 95% confidence interval (CI). Linear regression and correlation analyses, using Pearson’s coefficient (r), was also performed between RTL as a continuous variable and age, body mass index (BMI) and waist circumference (WC).

**Table 1:** The association of RTL with baseline characteristics and lifestyle.

		RTL			
		<1.06 (n=166)	1.06 - 1.432 (n=165)	>1.432 (n=166)	p-value
**Age (years)**	**Mean (±SD)**	48.56 ± 14.75	44.70 ± 15.01	42.92 ± 15.03	0.002
	<**40**	43 (25.9)^1^	61 (37.0)	75 (45.2)	0.009
	**40-60**	91 (54.8)	78 (47.3)	67 (40.42)	
	>**60**	32 (19.3)	26 (15.8)	24 (14.5)	
**Gender**	Female	105 (63.3)	106 (64.2)	108 (65.1)	0.94
**Marital status**	**Married**	106 (63.9)	114 (69.1)	109 (65.7)	0.17
	**Single**	28 (16.9)	33 (20.0)	37 (22.3)	
	**Other^2^**	32 (19.3)	18 (10.9)	20 (12.0)	
**Income**	<**600$**	58 (35.4)	48 (29.4)	46 (27.7)	0.31
	**600-999.9$**	58 (35.4)	55 (33.7)	56 (33.7)	
	≥**1000 $**	35 (21.3)	41 (25.2)	52 (31.3)	
	**I don’t know/no answer**	13 (7.9)	19 (11.7)	12 (7.2)	
**Education**	**No schooling or primary school**	74 (44.6)	55 (33.3)	52 (31.7)	0.03
	**Intermediate school**	30 (18.1)	56 (33.9)	48 (29.3)	
	**Secondary school or technical diploma**	43 (25.9)	39 (23.6)	44 (26.8)	
	**University degree**	19 (11.4)	15 (9.1)	20 (12.2)	
**Crowding index**	**Mean (±SD)**	1.52 ± 0.84	1.55 ± 0.90	1.50 ± 0.91	0.86
**Current smoker**		104 (62.7)	112 (67.9)	103 (62.0)	0.48
**Current cigarette smoker**		74 (44.6)	77 (46.7)	63 (38.0)	0.25
**Current narghileh smoker**		46 (27.7)	44 (26.7)	51 (30.7)	0.70
**Current Alcohol Drinker**		34 (20.5)	35 (21.2)	26(15.7)	0.38
**Coffee Drinker**		133 (80.1)	136 (82.4)	130 (78.3)	0.64
**BMI (kg/m^2^)**	**Mean (±SD)**	29.96 ± 5.73	28.92 ± 5.65	28.40 ± 5.94	0.045
**BMI (kg/m^2^)- categorical**	≥**30**	76 (45.8)	69 (41.8)	62 (37.3)	0.30
**Waist circumference (cm)**	**Mean (±SD)**	98.91 ± 14.46	95.87 ± 17.52	92.75 ± 14.12	0.001
**Body fat (Kg)**	**Mean (±SD)**	30.29 ± 11.28	28.24 ± 11.09	27.30 ± 12.06	0.05
**Muscle mass (Kg)**	**Mean (±SD)**	26.45 ± 6.80	26.24 ± 6.24	26.19 ± 6.19	0.93
**Levels of physical activity**	**Low-intensity activity**	76 (45.8)	82 (49.7)	80 (48.2)	0.97
	**Moderate-intensity activity**	54 (32.5)	49 (29.7)	51 (30.7)	
	**High-intensity activity**	36 (21.7)	34 (20.6)	35 (21.1)	
**Physical activity**	**None**	29 (17.5)	26 (15.8)	24 (14.5)	0.75
	**Any**	137 (82.5)	139 (84.2)	142 (85.5)	
**Number of hours sleep per night on weekdays**	≤**4 hours**	26 (15.7)	18 (10.9)	23 (13.9)	0.26
	**5-6 hours**	43 (25.9)	47 (28.5)	40 (24.1)	
	**6-7 hours**	50 (30.1)	37 (22.4)	44 (26.5)	
	**7-8 hours**	21 (12.7)	37 (22.4)	34 (20.5)	
	**8-9 hours**	16 (9.6)	13 (7.9)	19 (11.4)	
	≥**9 hours**	10 (6.0)	13 (7.9)	6 (3.6)	
**Number of hours sleep per night on weekend**	≤**4 hours**	23 (13.9)	20 (12.1)	21 (12.7)	0.80
	**5-6 hours**	30 (18.1)	32 (19.4)	30 (18.1)	
	**6-7 hours**	37 (22.3)	31 (18.8)	31 (18.7)	
	**7-8 hours**	26 (15.7)	35 (21.2)	37 (22.3)	
	**8-9 hours**	20 (12.0)	23 (13.9)	27 (16.3)	
	≥**9 hours**	30 (18.1)	24 (14.5)	20 (12.0)	
**Feel that you are not getting enough sleep**	**Never**	61 (37.4)	60 (37.0)	57 (35.2)	0.83
	**Rarely/sometimes/Frequently**	48 (29.4)	40 (24.7)	46 (28.4)	
	**Almost always**	54 (33.1)	62 (38.3)	59 (36.4)	
**Sleep difficulties**	**Never**	32 (19.3)	40 (24.2)	50 (30.1)	0.03
	**Rarely/sometimes/Frequently**	63 (38.0)	42 (25.5)	50 (30.1)	
	**Almost always**	71 (42.8)	83 (50.3)	66 (39.8)	

1. N (%)

2. Widow, divorced, or engaged

## RESULTS

### Relative telomere length

DNA for RTL was available for 497 out of the 501 recruited participants. Mean ± SD RTL was 1.42±0.83. The intra-assay geometric mean of the coefficients of variation for the telomere and single copy gene Ct values were less than 1% for both with a mean ± SD of 7 different assays of 0.92±0.17% and 0.58±0.11% for telomere and the single copy gene, respectively. As for inter-run reproducibility, there was a statistically significant high correlation between the RTL of 18 samples that were run on two different occasions (r= 0.88; P< 0.0001); in addition, the inter-assay geometric mean of the coefficient of variation was 6.49%.

**Figure 1.**
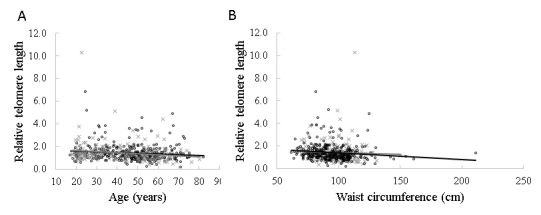
Correlation of relative telomere length with age and waist circumference Scatter plots showing the correlation of age (**A**) and waist circumference (**B**) with the relative telomere length of peripheral leucocyte blood respectively in males and females. The grey line and (×) represent the males (n=178) and the black line and (o) represent the females (n=319). The *P*-values were calculated from the linear regression analyses of the relationships between age, waist circumference and RTL in males and females, and the Pearson correlation was used.

**Table 2:** The association of RTL with chronic diseases and laboratory measurements.

		RTL			
		<1.06 (n=166)	1.06 - 1.432 (n=165)	>1.432 (n=166)	p-value
Diabetes					
Definite diabetes^2^		25 (15.1)^1^	30 (18.2)	20 (12.0)	0.30
Self-reported diabetes or hyperglycemia diagnosis		24 (14.5)	27 (16.4)	13 (7.8)	0.05
Diabetes treatment		26 (15.7)	23 (13.9)	12 (7.2)	0.05
Fasting blood sugar (mg/dL)	Abnormal	90 (54.2)	81 (49.1)	76 (45.8)	0.30
Insulin (µIU/mL)	Mean (±SD)	28.28 ± 11.10	29.68 ± 18.59	28.29 ± 19.46	0.74
HbA1C (%)	Mean (±SD)	5.97 ± 1.38	6.02 ± 1.38	5.80 ± 1.30	0.32
C peptide (ng/dL)	Mean (±SD)	3.34 ± 1.51	3.01 ± 1.28	3.01 ± 1.59	0.07
Hypertension (HTN)					
Definite HTN^3^		73 (44.2)	61 (37.0)	47 (28.3)	0.01
Self-reported HTN diagnosis		56 (33.7)	39 (23.6)	23 (13.9)	<0.0001
HTN treatment		52 (31.3)	36 (21.8)	22 (13.3)	<0.0001
Systolic blood pressure (mm/Hg)	Mean (±SD)	122.38 ± 17.82	123.01 ± 21.22	119.41 ± 18.34	0.19
Diastolic blood pressure (mm/Hg)	Mean (±SD)	75.38 ± 9.31	74.95 ± 10.81	73.94 ± 9.76	0.40
Dyslipidemia					
Self-reported dyslipidemia diagnosis		53 (31.9)	35 (21.2)	31 (18.7)	0.01
Dyslipidemia treatment		43 (25.9)	28 (17.0)	23 (13.9)	0.02
HDL (mg/dL)	Mean (±SD)	50.04 ± 15.02	49.47 ± 14.29	49.45 ± 15.22	0.92
LDL (mg/dL)	Mean (±SD)	110.64 ± 35.45	111.65 ± 42.53	104.08 ± 34.34	0.14
Triglycerides (mg/dL)	Mean (±SD)	142.23 ± 70.39	151.04 ± 141.95	131.12 ± 79.07	0.21
Metabolic Syndrome					
Metabolic Syndrome^4^		90 (54.2)	79 (47.9)	85 (51.2)	0.51
Atherosclerotic cardiovascular disease (ASCVD) 10yrs Risk					
ASCVD 10yrs Risk (%)^5^	Mean (±SD)	7.82 ± 11.72	7.82 ± 14.27	4.94 ± 7.03	0.04
Others					
CRP (mg/L)	Mean (±SD)	13.07 ± 8.80	13.01 ± 13.35	10.28 ± 6.89	0.02
Cortisol (µg/dL)	Mean (±SD)	18.35 ± 10.43	19.14 ± 13.95	16.89 ± 10.59	0.22

1. N (%)

2. Subjects who were diagnosed with diabetes and/or both fasting blood sugar (FBS) was ≥126 mg/dL and HbA1C was ≥6.5%.

3. Subjects who were diagnosed with hypertension or had an abnormal blood pressure reading upon recruitment (systolic blood pressure≥140 mm/Hg or diastolic blood pressure≥90 mm/Hg).

4. Definition was based on Alberti et al. Circulation 2009.

5. Definition was based on the 2013 ACC/AHA guideline.

### Associations with relative telomere length

As shown in Figure 1, there was a statistically significant correlation between age and RTL [r=-0.14; *P*=0.002], as well as WC and RTL [r=-0.09; *P*=0.035]. This was not the case for BMI (r=-0.06; *P*=0.17]).

Similarly, and as shown in Table 1, older age (*P*=0.002 for continuous and *P*=0.009 for categorical) and wider WC (*P*=0.001) were statistically significantly associated with shorter RTL. A similar statistically significant association was found with BMI (*P*=0.045). In addition, lower education levels were associated with significantly shorter RTL (*P*=0.03), and different levels of sleep difficulties were associated with statistically significant differences in RTL (*P*=0.03).

As for the association of RTL with outcome (disease and abnormal laboratory values), and as seen in Table 2, participants with definite hypertension and those who were known to have hypertension or treated for it had a statistically significant shorter RTL than those who were not (*P* = 0.01; <0.0001 and <0.0001, respectively). Similarly, those who were known to have dyslipidemia, being treated for dyslipidemia or had a higher fasting blood cholesterol levels had a statistically significant shorter RTL (*P* = 0.01; 0.02 and 0.03, respectively). No statistically significant associations were found with diabetes; there wet yet trends of shorter RTL in subjects who were on treatment for diabetes (*P*=0.05), and those who had higher C-peptide levels (*P*=0.07). In addition, there was no statistically significant association with MetS; yet participants who were at a relatively lower risk of developing cardiovascular diseases in 10 years (risk (%): 4.94±7.03) had statistically significantly longer RTL (*P*=0.04). Interestingly, higher CRP levels were statistically significantly associated with shorter RTL (*P*=0.02).

**Table 3:** Stepwise multinomial logistic regression of potentially significant predictors of RTL including waist circumference as a marker for obesity with RTL

	RTL				
	≤1.060		1.060 - 1.432		>1.432
Variables	OR ( 95 % CI )	P-value	OR ( 95 % CI )	P-value	
Education - Intermediate school	0.50 (0.28 - 0.89)	0.02	1.22 (0.71 - 2.11)	0.47	Reference
Education - Secondary school/technical diploma	0.84 (0.47 - 1.48)	0.55	1.02 (0.57 - 1.83)	0.95	Reference
Education - University degree	0.98 (0.45 - 2.11)	0.96	0.99 (0.44 - 2.23)	0.99	Reference
Waist circumference -WC (cm)	1.30 (1.11 - 1.52)	0.001	1.17 (1.00 - 1.37)	0.05	Reference
Any Sleeping Difficulty - Rarely, sometimes, or frequently	2.01 (1.11 - 3.62)	0.02	1.04 (0.58 - 1.88)	0.88	Reference
Any Sleeping Difficulty - Almost always	1.73 (0.97 - 3.08)	0.06	1.55 (0.91 - 2.67)	0.11	Reference

Age (reference: <40); Education (reference: No schooling/primary school); Waist circumference (per unit increase of 10); Any sleeping Difficulty (reference: Never). Body mass index -BMI was not included due to collinearity with WC.

As seen in the multinomial logistic regression in Table 3, only the following potential predictors were retained in the model: level of education, sleeping difficulties, and WC. For instance, the odd for subjects with intermediate schooling to have a statistically significantly shorter RTL was lower when compared to those with no or only primary schooling [OR (95% CI): 0.50 (0.28-0.89) in the first RTL tertile]. In addition, subjects who had some sleeping difficulty had a statistically significantly shorter RTL when compared to those with no sleeping difficulties at all [OR (95% CI): 2.01 (1.11-3.62) in the first RTL tertile]. Importantly, statistically significantly shorter RTL was found with every additional 10 cm of WC [OR (95% CI): 1.30 (1.11-1.52) for first RTL tertile]. A similar analysis was done with BMI instead of WC (Supplementary Table 1), and only age, education and sleeping difficulties were retained in the model with similar direction of results as with WC.

In addition, and after performing the multivariate logistic regression and adjusting for the “predictors” that remained statistically significantly associated with RTL (age, education, WC and sleeping difficulties), diagnosis and treatment of hypertension were retained in the model whereby the odds of having hypertension or being treated for hypertension were higher in patients who had shorter RTL. This was statistically significant in the first RTL tertiles [OR (95% CI): 2.45 (1.36-4.44) and 2.28 (1.22-4.26), respectively] with a similar trend, though not statistically significant, in the second RTL tertiles. Higher CRP levels were found to be statistically significantly associated with shorter RTL in the second tertile though there was no similar trend in the first RTL tertile (Table 4).

## DISCUSSION

To our knowledge, this is the first study in Lebanon to show an association between age, central obesity and hypertension on RTL in peripheral blood, and the first in the Arab region to show the association between sleep difficulties and RTL. These results are important from an ethnic perspective knowing that RTL may be differentially affected in different ethnicities [44, 45].

Telomeres are known to shorten with age due to the end replication problem [16]; therefore, the extent of telomere shortening was proposed to play an important role in determining biological age, and to be a better indicator than chronological age for defining the health and well-being of an individual [46, 47]. Accordingly, and similarly to our results, several studies showed that telomere shortening is progressive with aging and associated with age-related diseases. For example, in a study done on elderly people aged 60 years and older, telomere shortening was found to be associated with increased mortality rates in some age-related diseases including cardiovascular diseases [8]. In another study done on Arab patients, short RTL was associated with increased prevalence of asymptomatic coronary atherosclerosis [32]. As for gender, and similar to other studies [47, 48], our results did not show any difference in RTL between men and women. Although it was previously found that, in later adulthood, men tend to have shorter telomere length than females [49].

**Table 4:** Stepwise multivariate logistic regression of RTL tertiles with chronic disease and laboratory values with and without adjustment with potentially significant predictors of RTL^1^

	≤1.060 (n=166)	P-value	RTL 1.060 - 1.432 (n=165)	P-value	>1.432 (n=166)
Diabetes					
Definite diabetes					
*Cases, n (%)*	25 (15.1)		30 (18.2)		20 (12.0)
*Unadjusted*	1.29 (0.69 - 2.43)	0.42	1.62 (0.88 - 2.99)	0.12	Reference
*Adjusted*	0.76 (0.38 - 1.52)	0.43	1.29 (0.65 - 2.56)	0.46	Reference

Diabetes diagnosis					
*Cases, n (%)*	24 (14.5)		27 (16.4)		13 (7.8)
*Unadjusted*	1.99 (0.97 - 4.06)	0.06	2.30 (1.14 - 4.64)	0.02	Reference
*Adjusted*	1.31 (0.61 - 2.81)	0.48	1.94 (0.91 - 4.13)	0.09	Reference

Diabetes treatment					
*Cases, n (%)*	26 (15.7)		23 (13.9)		12 (7.2)
*Unadjusted*	2.38 (1.16 - 4.90)	0.02	2.08 (1.00 - 4.33)	0.05	Reference
*Adjusted*	1.67 (0.78 - 3.56)	0.19	1.71 (0.79 - 3.73)	0.17	Reference

Abnormal fasting blood sugar					
*Cases, n (%)*	90 (54.2)		81 (49.1)		76 (45.8)
*Unadjusted*	1.40 (0.91 - 2.16)	0.12	1.14 (0.74 - 1.76)	0.55	Reference
*Adjusted*	0.92 (0.57 - 1.50)	0.74	0.96 (0.59 - 1.56)	0.88	Reference

Insulin (µIU/mL)					
*Mean, SD*	28.28 ± 11.10		29.68 ± 18.59		28.29 ± 19.46
*Unadjusted*	-0.01 (-4.03; 4.02)	1.00	1.40 (-2.66; 5.46)	0.50	Reference
*Adjusted*	-2.34 (-6.20; 1.52)	0.23	-0.06 (-3.92; 3.81)	0.98	Reference

HbA1C (%)					

*Mean, SD*	5.97 ± 1.38		6.02 ± 1.38		5.80 ± 1.30
*Unadjusted*	0.16 (-0.13; 0.45)	0.29	0.22 (-0.08; 0.51)	0.15	Reference
*Adjusted*	-0.10 (-0.37; 0.17)	0.48	0.09 (-0.18; 0.36)	0.50	Reference

C-peptide (ng/dL)					
*Mean, SD*	3.34 ± 1.51		3.01 ± 1.28		3.01 ± 1.59
*Unadjusted*	0.33 (0.0; 0.64)	0.04	0.002 (-0.32; 0.32)	0.99	Reference
*Adjusted*	0.09 (-0.20; 0.38)	0.55	-0.12 (-0.41; 0.17)	0.41	Reference
Hypertension (HTN)					
Definite HTN					
*Cases, n (%)*	73 (44.2)		61 (37.0)		47 (28.3)
*Unadjusted*	2.01 (1.27 - 3.17)	0.003	1.48 (0.93 - 2.36)	0.09	Reference
*Adjusted*	1.42 (0.85 - 2.38)	0.18	1.31 (0.77 - 2.23)	0.31	Reference

HTN diagnosis					
*Cases, n (%)*	56 (33.7)		39 (23.6)		23 (13.9)
*Unadjusted*	3.16 (1.83 - 5.46)	<0.0001	1.92 (1.09 - 3.40)	0.02	Reference
*Adjusted*	2.45 (1.36 - 4.44)	0.003	1.71 (0.92 - 3.19)	0.09	Reference

HTN treatment					
*Cases, n (%)*	52 (31.3)		36 (21.8)		22 (13.3)
*Unadjusted*	2.99 (1.71 - 5.20)	<0.0001	1.83 (1.02 - 3.27)	0.04	Reference
*Adjusted*	2.28 (1.22 - 4.26)	0.01	1.61 (0.83 - 3.11)	0.15	Reference

Systolic blood pressure (mm/Hg)					
*Mean, SD*	122.38 ± 17.82		123.01 ± 21.22		119.41 ± 18.34
*Unadjusted*	2.97 (-1.18; 7.12)	0.16	3.60 (-0.54; 7.75)	0.09	Reference
*Adjusted*	-0.20 (-3.95; 3.56)	0.25	2.17 (-1.54; 5.88)	0.25	Reference

Diastolic blood pressure (mm/Hg)					
*Mean, SD*	75.38 ± 9.31		74.95 ± 10.81		73.94 ± 9.76
*Unadjusted*	1.44 (-0.72; 3.60)	0.19	1.01 (-1.15; 3.17)	0.36	Reference
*Adjusted*	-0.20 (-2.21; 1.82)	0.85	0.28 (-1.71; 2.27)	0.78	Reference

Dyslipidemia					
Dyslipidemia diagnosis					
*Cases, n (%)*	53 (31.9)		35 (21.2)		31 (18.7)
*Unadjusted*	2.04 (1.23 - 3.40)	0.01	1.17 (0.68 - 2.01)	0.56	Reference
*Adjusted*	1.42 (0.82 - 2.47)	0.21	0.94 (0.52 - 1.70)	0.84	Reference

Dyslipidemia treatment					
*Cases, n (%)*	43 (25.9)		28 (17.0)		23 (13.9)
*Unadjusted*	2.17 (1.24 - 3.81)	0.01	1.27 (0.70 - 2.31)	0.43	Reference
*Adjusted*	1.59 (0.87 - 2.91)	0.13	1.03 (0.54 - 1.96)	0.92	Reference

HDL (mg/dL)					
*Mean, SD*	50.04 ± 15.02		49.47 ± 14.29		49.45 ± 15.22
*Unadjusted*	0.59 (-2.61 ; 3.79)	0.72	0.02 (-3.19 ; 3.23)	0.99	Reference
*Adjusted*	2.05 (-1.10 ; 5.19)	0.20	0.76 (-2.36 ; 3.88)	0.63	Reference

LDL (mg/dL)					
*Mean, SD*	110.64 ± 35.45		111.65 ± 42.53		104.08 ± 34.34
*Unadjusted*	6.56 (-1.55 ; 14.67)	0.11	7.57 (-0.56 ; 15.70)	0.07	Reference
*Adjusted*	2.65 (-5.40 ; 10.70)	0.52	6.56 (-1.40 ; 14.53)	0.11	Reference

Triglycerides (mg/dL)					
*Mean, SD*	142.23 ± 70.39		151.04 ± 141.95		131.12 ± 79.07
*Unadjusted*	11.11 (-10.92 ; 33.14)	0.32	19.92 (-2.14 ; 41.98)	0.08	Reference
*Adjusted*	3.68 (-17.93 ; 25.29)	0.74	13.42 (-7.94 ; 34.78)	0.22	Reference

Metabolic syndrome^2^					
*Cases, n (%)*	90 (54.2)		79 (47.9)		85 (51.2)
*Unadjusted*	1.13 (0.73 - 1.74)	0.58	0.87 (0.57 - 1.35)	0.54	Reference
*Adjusted*	0.87 (0.55 - 1.39)	0.57	0.75 (0.47 - 1.19)	0.22	Reference

Atherosclerotic cardiovascular disease (ASCVD) 10yrs Risk^3^					
*Mean, SD*	7.82 ± 11.72		7.82 ± 14.27		4.94 ± 7.03
*Unadjusted*	2.88 (0.34 ; 5.42)	0.03	2.88 (0.36 ; 5.41)	0.02	Reference
*Adjusted*	1.54 (-0.95 ; 4.04)	0.22	2.39 (-0.05 ; 4.82)	0.05	Reference

Other					

CRP (mg/L)					
*Mean, SD*	13.07 ± 8.80		13.01 ± 13.35		10.28 ± 6.89
*Unadjusted*	2.78 (0.62 ; 4.95)	0.01	2.73 (0.56 ; 4.90)	0.01	Reference
*Adjusted*	1.78 (-0.33 ; 3.89)	0.10	2.38 (0.28 ; 4.49)	0.03	Reference

Cortisol (µg/dL)					
*Mean, SD*	18.35 ± 10.43		19.14 ± 13.95		16.89 ± 10.59
*Unadjusted*	1.46 (-1.10 ; 4.03)	0.26	2.25 (-0.34 ; 4.84)	0.09	Reference
*Adjusted*	1.94 (-0.62 ; 4.51)	0.14	2.53 (-0.04 ; 5.11)	0.05	Reference

1. Stepwise regression with the following covariates: Age (reference: <40); Education (reference: No schooling/primary school); WC (per unit increase of 10); Any sleeping Difficulty (reference: Never).

2. Stepwise regression with the following covariates: Age (reference: <40); Education (reference: No schooling/primary school); Any sleeping Difficulty (reference: Never).

3. Stepwise regression with the following covariates: Education (reference: No schooling/primary school); WC (per unit increase of 10); Any sleeping Difficulty (reference: Never).

In the current study, and similarly to others [21, 31, 32, 50, 51], we have shown that people with wider WC had shorter telomere length. Moreover, in our study, wider WC remained to be associated with short RTL after adjusting for other predictors in the multinomial logistic regression analysis, whereas the significance of BMI was lost. In addition, and after adjusting for WC among other potential predictors, short telomere length was associated with hypertension, a finding that has been previously reported [12, 31, 52]. WC is a measure of abdominal obesity, and it is known to be a better predictor of cardiovascular diseases than BMI [6-8]. For instance, a cross-sectional study done on young Canadian adults showed that WC was better than BMI in predicting the blood lipid profile which is a CVD risk factor [53]. In addition, WC was found to be a better predictor of obesity-related CVD risk, as people with different BMI categories, but similar WC, were shown to have the same risk [54, 55]. In contrast to WC, BMI only reflects the body size and total body fat, yet it does not reflect fat distribution [56]; nevertheless, WC does not estimate fat body composition and this could be the reason behind some studies not being able to show an association between WC and CVD risk factors [57-59].

Interestingly in this study, sleeping difficulties, but not short duration, were associated with shorter RTL. Although Prather et al. showed similar results [60], others revealed that short durations of sleep were also associated with short telomere length [61, 62]. It is important to note that people self-reporting and describing their sleep may not be accurate as it is subject to their current mood and recall. Moreover, one can argue that sleep quality decreases with increasing age; hence the correlation between short RTL and sleep difficulties may be a reflection of the age effect. In this study, we also found that intermediate education is significantly associated with longer RTL when compared to no schooling, and this was reported by Adler *et al*. [63].

The reason behind the associations between WC, hypertension, sleep and RTL may be attributed to the increased level of oxidative stress that alters telomere length and accelerates aging [64, 65]. For instance, on one hand, it is known that coronary heart diseases are associated with increased oxidative stress production and inflammation which may be contributing to shorter telomere length [66-68]. In addition, Sampson et al. found that monocytes telomere length was shorter in patients with type 2 diabetes mellitus, and this was associated with high oxidative DNA damage when compared to controls [69]. Moreover, WC was previously found to be associated with increased levels of circulating oxidized LDL and CRP that may contribute to increased oxidative stress [70]. On the other hand, antioxidants may decrease the rate of the telomeric loss [71]. For instance, exercise was shown to reduce telomere shortening probably due to a decrease in oxidative stress or increase in the oxidative stress defense [72]. As for sleep, it was previously shown that poor sleep quality is linked to increased cortisol and cytokines secretion and reduction in melatonin, an anti-inflammatory sleep regulator, all of which might affect telomere length through increased oxidative stress [73-76].

In this study, we unfortunately did not measure markers for oxidative stress and hence cannot deduce whether it is the driver behind our findings. Nevertheless, one may look at CRP as a marker of inflammation and potentially oxidative stress, especially that in our study, and similarly to Attas *et al*. [31], high CRP levels were associated with shorter RTL. CRP is an important indicator of inflammation and a clinically useful biomarker for CVD risk including hypertension risk [77, 78], and as a matter of fact in our study, patients who were diagnosed with hypertension had statistically significantly higher mean levels of CRP(mg/L) (14.90±13.05) when compared to those who did not (10.58±7.56) (*P*<0.001). As for sleep difficulties, and although in our study there was no statistically significant association between CRP and sleep difficulties (*P*=0.182), multinomial logistic regression of the associations of sleep difficulty and CRP with RTL retained both variables in the model whereby any sleeping difficulty and higher levels of CRP were both statistically significantly associated with shorter RTL (data not shown), meaning that inflammation must have contributed to the resulting telomere length shortening. In addition, when running a multinomial logistic regression of sleeping difficulty with age, WC and hypertension, some sleeping difficulty remained statistically significantly associated with shorter RTL (first tertile) with OR (95% CI) of 1.94 (1.08-3.50) and 1.99 (1.10-3.59) with and without adding CRP levels in the model respectively. These results warrant further evaluation in parallel to oxidative stress markers such as hydrogen peroxide concentrations in peripheral blood as recently performed by Kim et al. in the setting of obstructive sleep apnea [79].

This study suffers from some additional limitations. For instance, it is based on a cross sectional design whereby the causes behind our results need to be clarified. Furthermore, although we controlled for different variables such as age, WC, education, and sleep, the presence of other residual and uncollected factors that may influence telomere length is possible. Although the prevalence of MetS was high in this cohort, the absence of an association with RTL was probably driven by the fact that diabetes, HDL and triglyceride levels were not statistically significantly associated with RTL. It is unfortunate that, although there was a statistically significant association between RTL and 10-year ASCVD, this association was lost after adjustment for confounders. This is probably because most of the participants had a relatively low estimated risk (Mean ± SD for the whole cohort: 6.80 ± 11.37). In addition, this study is exploratory in nature with no power analysis; therefore, the sample size may have been insufficient to show statistically significant associations with variables such as the 10-year ASCVD. Finally, it is still unclear why education is related to RTL, though it may be speculated that educated individuals are exposed to less stress due to better financial and social security.

In conclusion, we showed that older individuals with wider WC and sleep difficulties have shorter RTL in their peripheral blood. Moreover, shorter RTL is associated with increased risk of hypertension after adjusting for predictors. It is hoped that telomere length measurement be potentially used as a biomarker for biological age and age-related cardiovascular diseases and progression in the Lebanese population. These results need to be further studied to be able to understand the residing causes behind such telomere length alteration.

## 

**Supplementary Table 1:** Stepwise multinomial logistic regression of potentially significant predictors of RTL including body mass index as a marker for obesity with RTL

	RTL				
	≤1.060		1.060 - 1.432		>1.432
Variables	OR (95 % CI )	P-value	OR (95 % CI)	P-value	
Age - 40-60 years	2.41 (1.45 - 4.02)	0.001	1.35 (0.84 - 2.19)	0.22	Reference
Age - >60 years	2.12 (1.06 - 4.23)	0.03	1.25 (0.63 - 2.48)	0.52	Reference
Education - Intermediate school	0.49 (0.27 - 0.88)	0.02	1.19 (0.68 - 2.06)	0.54	Reference
Education - Secondary school or technical diploma	0.88 (0.49 - 1.58)	0.67	1.00 (0.55 - 1.82)	1.00	Reference
Education - University degree	0.96 (0.45 - 2.06)	0.91	0.90 (0.40 - 2.01)	0.80	Reference
Any sleeping difficulty - Rarely, sometimes, or frequently	2.04 (1.13 - 3.69)	0.02	1.04 (0.58 - 1.88)	0.89	Reference
Any sleeping difficulty - Almost always	1.61 (0.90 - 2.86)	0.11	1.49 (0.87 - 2.55)	0.15	Reference

Age (reference: <40); Education (reference: No schooling/primary school); Body mass index -BMI (per unit increase of 1); Any sleeping Difficulty (reference: Never). Waist Circumference (WC) was not included due to collinearity with BMI.
